# The impact of brominated flame retardants (BFRs) on pulmonary function in US adults: a cross-sectional study based on NHANES (2007–2012)

**DOI:** 10.1038/s41598-024-57302-9

**Published:** 2024-03-18

**Authors:** Haiyan Mao, Tong Lin, Shanshan Huang, Zhenye Xie, Shaofeng Jin, Xingkai Shen, Yuhong Jin, Yi Ding

**Affiliations:** Department of Critical Care Medicine, Ningbo Medical Center Lihuili Hospital, Ningbo, 315100 China

**Keywords:** Brominated flame retardants, Pulmonary function, Ventilatory dysfunction, Cross-sectional study, Diseases, Medical research, Risk factors

## Abstract

Brominated flame retardants (BFRs) are a group of chemicals widely used in various applications to prevent or slow down the spread of fire. However, they have adverse effects on human health. There is a relative scarcity of population-based studies regarding BFRs, particularly their impact on the respiratory system. This study aimed to investigate the influence of BFRs on pulmonary function using data from the National Health and Nutrition Examination Survey. The study found that elevated serum concentrations of certain BFRs were associated with pulmonary ventilatory dysfunction. Adjusted analyses revealed positive correlations between PBDE47, PBDE183, and PBDE209 concentrations and ventilatory dysfunction. The analysis of mixed BFRs showed a positive relationship with pulmonary ventilation dysfunction, with PBDE47 making the most significant contribution. Our study demonstrates that both individual and combined BFRs exposure can lead to impaired pulmonary ventilation function. These findings provide evidence of the adverse effects of BFRs on lung function, emphasizing the importance of further investigating the potential health consequences of these compounds. Further large-scale longitudinal studies are needed to investigate this relationship in the future.

## Introduction

Polybrominated diphenyl ethers (PBDEs) and 2,2′,4,4′,5,5′-hexabromobiphenyl (PBB-153) represent a category of brominated flame retardants. Due to their excellent heat stability and high fire-retardant efficiency, they find widespread application in various domains such as textiles, furniture, construction materials, electronic products, and automotive interiors^1,2^. BFRs, characterized by their lipophilicity, persistence, and environmental ubiquity, are extensively present in air, water, soil, marine animals, terrestrial mammals, and even human breast milk^3,4^. Although some of the current BFRs have been classified as persistent organic pollutants (POPs) by the Stockholm Convention, an international treaty administered by the United Nations Environment Programme, and their applications have been restricted and gradually phased out, they have been consistently detected in consumer durables, foodstuffs, and house dust, and are still a hot topic in the current research on environmental science^5^. Previous studies have demonstrated that BFRs possess various toxic effects, including endocrine disruption, liver toxicity, kidney toxicity, immune toxicity, neurotoxicity, reproductive and developmental toxicity, and carcinogenicity, posing significant threats to both animal and human health^6–11^.

Respiratory system diseases represent one of the most pronounced public health and medical issues globally, especially following the COVID-19 pandemic in 2019. Chronic respiratory diseases rank among the "big four chronic diseases," being one of the leading causes of disease burdens globally, with chronic obstructive pulmonary disease ranking as the third leading cause of death worldwide. According to World Health Organization statistics, the global number of deaths due to chronic respiratory diseases increased by 4.1 million in 2019, with the highest age-standardized mortality rate showing a decrease of approximately 37%. Respiratory diseases have had a significant impact on the world economy, including medical costs, loss of productivity, and social welfare expenditures^12–15^. With the increasing prominence of environmental pollution, a substantial population of smokers, and aging populations, the prevention and control of respiratory system diseases are becoming increasingly severe.

Certain brominated flame retardants, owing to their semi-volatile nature, slowly release into the air over time or adhere to airborne particles, causing an impact on the atmospheric environment^16,17^. Reportedly, the concentration of PBDEs in outdoor air in the United States varies from 5 to 100 µg per cubic meter^18^. Research indicates that brominated flame retardants can exist in the respiratory tracts of both animals and humans, influencing bronchial epithelium, including inhibiting cell vitality, promoting cell apoptosis, inducing cellular DNA damage, and fostering inflammation and oxidative stress, consequently leading to decreased pulmonary function^19–23^. Human exposure to BFRs occurs primarily through various pathways, such as dust ingestion, air inhalation (especially at home or in electronic industries), diet, and skin contact with dust^24,25^. In the United States, the estimated dietary intake of PBDEs is around 50 ng/day, mainly sourced from the consumption of dairy, meat, and fish^26^. Studies indicate a positive correlation between the concentration of BFRs through inhalation and skin contact pathways and the serum concentration of BFRs in the human body^27–29^. Due to factors such as individual habits, occupation, dietary preferences, variations in BFR concentrations between rooms, the use of electronic products, and the use of consumer goods, there is considerable uncertainty in estimating BFR exposure levels^11,30^. The concentration of flame retardants in human serum serves as a valuable indicator for assessing human exposure to BFRs and related health risks^31,32^.

Over the past forty years, human exposure to brominated flame retardants has been on the rise, yet there remains a scarcity of data concerning population-based studies on BFRs, particularly those investigating their impact on the respiratory system. This paper aims to explore the influence of brominated flame retardants on human pulmonary function by gathering relevant data from the NHANES database, thereby would help to enhance public awareness of prevention to the BFRs exposures.

## Materials and methods

### Study population

**Figure 1:**
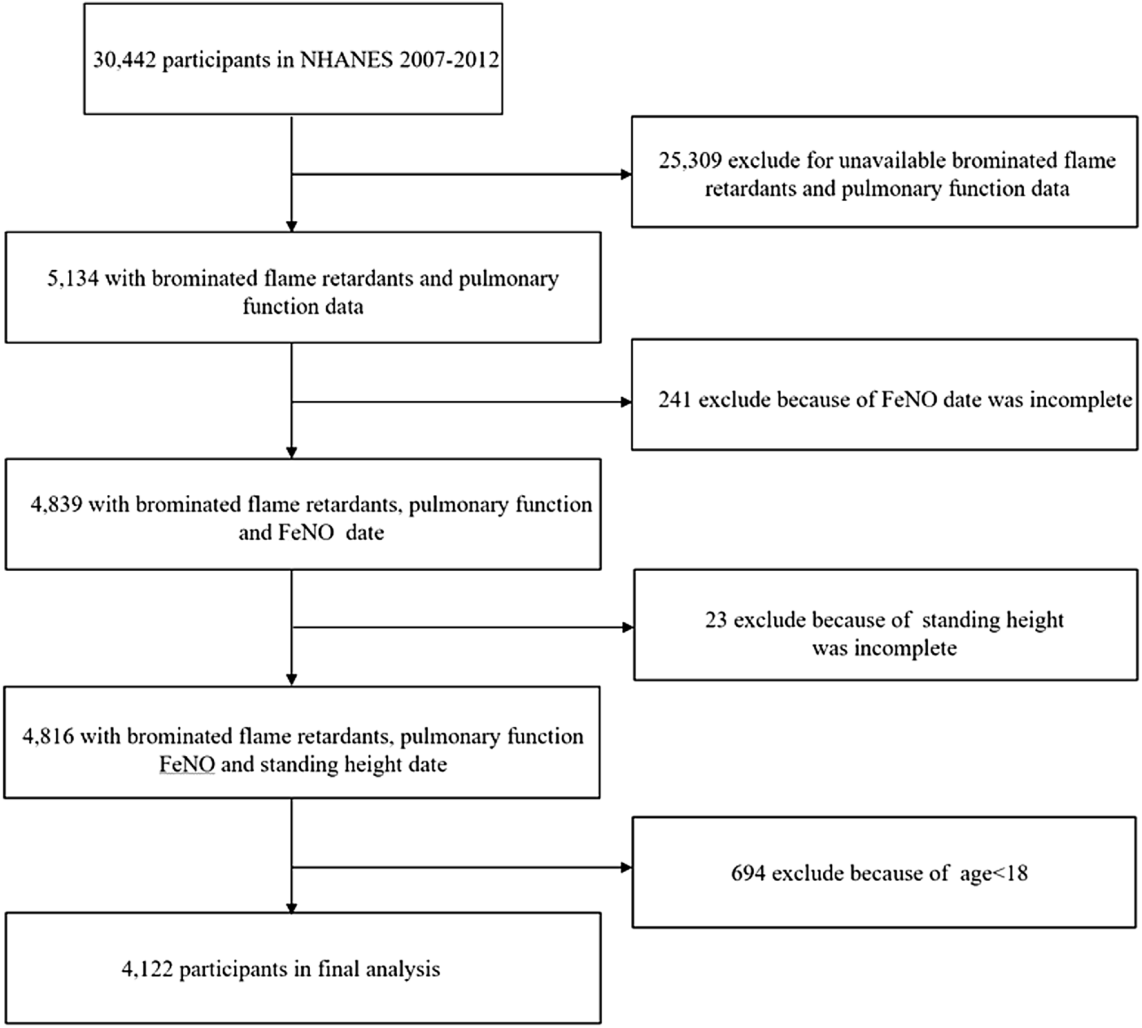
Screening conditions and process for the study population.

This cross-sectional study utilized data from the NHANES database, a nationwide survey conducted by the National Center for Health Statistics (NCHS) under the Centers for Disease Control and Prevention. The survey employed a complex, multi-stage sampling design to gather data representing the non-institutionalized U.S. population. Data from three NHANES cycles between 2007 and 2012 were analyzed due to the inclusion of pulmonary function tests only in these cycles. Over the survey period, 30,442 individuals participated. To focus the analysis, individuals under 18 years old and those lacking information on flame retardants, pulmonary function, or height were excluded, resulting in a final cohort of 4,122 participants, as illustrated in Fig. 1.

### Participant stratification

This study categorized the population into two groups: normal pulmonary function and pulmonary ventilatory dysfunction, based on the standardized Z-score for lung function as proposed by the American Thoracic Society (ATS). The Z-score indicates the number of standard deviations a measurement differs from the mean predicted value. Z-scores > − 1.645 were considered as indicating normal pulmonary function^33,34^. These scores were used instead of FEV1 predicted values% to assess the severity of lung function, mitigating differences arising from gender, age, height, and ethnicity. We utilized the prediction equations provided by the Global Lung Function Initiative (GLI-2012) to calculate FEV1 predicted values and Z-scores through specially developed software (GLI-2012 Data Conversion software; www.lungfunction.org/files/InstallGLI2012_DataConversion.EXE)^35^.

### Ethics considerations

The NCHS Research Ethics Review Board approved the NHANES program, and all participants gave their consent to join the survey. The NCHS allows researchers to use the data for research purposes. The NCHS anonymizes the NHANES data before releasing them and they remain anonymous during analysis. So, this study did not need extra ethical approval or informed consent for the secondary data analysis it did. Further details on the NCHS Research Ethics Review Board approval can be accessed on the NHANES website (https:// www.cdc.gov/nchs/nhanes/irba98.htm).

### Pulmonary function testing

Pulmonary function testing within NHANES was sponsored by several institutes including the National Heart, Lung, and Blood Institute of the National Institute of Health, the Centers for Disease Control and Prevention, the National Center for Health Statistics, and the National Institute for Occupational Safety and Health (NIOSH). The spirometry measurement procedures aligned with the recommendations of the American Thoracic Society. Age-eligible participants were enrolled in pulmonary function tests. Examinees were excluded if they currently were experiencing chest pain or physical issues, using supplemental oxygen, had undergone recent eye, chest, or abdominal surgery, experienced a heart attack, stroke, tuberculosis exposure, or had recently coughed up blood. Adults with a history of a detached retina or a collapsed lung and children with painful ear infections were also excluded. Baseline spirometry data from the initial test were utilized.

### Brominated flame retardants (BFRs) assessment

The NHANES Laboratory Procedures Manual provides comprehensive guidelines for collecting, storing, and processing blood specimens. Concentrations of brominated flame retardants were assessed using an automated liquid/liquid extraction and isotope dilution-ultra-performance liquid chromatography-tandem mass spectrometry method. The NHANES dataset included eleven polybrominated diphenyl ethers (PBDEs) comprising PBDE-17, PBDE-28, PBDE-47, PBDE-66, PBDE-85, PBDE-99, PBDE-100, PBDE-153, PBDE-154, PBDE-183, PBDE-209, and 2,2′,4,4′,5,5′-hexabromobiphenyl (PBB-153).

### Covariates

Several potential confounding variables were considered, including age, gender, race/ethnicity (Non-Hispanic Black, Non-Hispanic White, Mexican American, and Other), family poverty income ratio (FIR), and body mass index (BMI). A history of smoking was defined as smoking 100 or more cigarettes in one's lifetime. Alcohol use history was confirmed by ascertaining if participants had consumed 12 or more alcoholic drinks in a year. Histories of medical conditions were verified by asking participants if they had been diagnosed with diabetes or hypertension by a doctor. Additionally, FeNO, a non-invasive marker of airway inflammation, was included. Nitric Oxide (NO) is typically produced in the respiratory tract and can be detected in exhaled breath, showing higher levels in individuals with respiratory conditions like asthma and COPD^36–39^.

### Statistical analyses

Non-normally distributed continuous data were expressed using medians and interquartile ranges. Group comparisons were conducted using the Mann–Whitney U test or Kruskal–Wallis H test, whereas categorical data were presented as percentages or proportions and analyzed using the Chi-square test. To comprehensively assess the impact of BFRs on pulmonary function, survey-weighted generalized logistic regression models were employed to adjust variables and calculate OR values and their corresponding 95% confidence intervals. Considering the complex multi-stage sampling design in NHANES, in accordance with the guidance from the National Center for Health Statistics, this study employed the "sum of adjusted subsample B weights" or " adjust sum sampling weights in same pool". Given the non-normal distribution of BFRs and substantial numerical differences among indicators, a logarithmic transformation was applied. Four models were provided: Model 1 did not adjust for any covariates, Model 2 controlled for age and gender, Model 3 additionally adjusted for race, FIR, and BMI on Model 2, while Model 4 further adjusted for baseline medical history and smoking/alcohol history on Model 2. Additionally, subgroup interaction analyses were conducted based on age and gender.

To investigate the potential nonlinear association between individual BFR concentrations and impaired lung ventilatory function, this study employed restricted cubic splines (RCS) with three knots to explore the relationship between individual BFRs and pulmonary function. As BFRs comprise multiple substances, a Quantile G-computation (QGC) analysis was utilized to comprehensively assess the overall impact of BFRs exposure on pulmonary function. QGC is a straightforward and computationally efficient method for estimating the association between a combination of exposures and the desired health outcome. The training set (30%) and validation set (70%) are randomly allocated in this model.

All analyses were conducted in this study using R software version 4.3.1. (Core Team, Vienna, Austria), and a two-sided *P* value of < 0.05 was considered statistically significant.

### Ethical approval

This study was approved by the NCHS Research Ethics Review Board (ERB) and followed the ethical standards for human research. The details of the NCHS Research Ethics Review Board Approval can be found on the NHANES website (https:// www.cdc.gov/nchs/nhanes/irba98.htm).

### Patient and public involvement

Patients and/or the public were not involved in the design, or conduct, or reporting, or dissemination plans of this research.

## Results

### Characteristics of the study participants

The baseline characteristics of this study population are shown in Table 1. A total of 4122 eligible participants were recruited for this study, comprising 2,128 males and 1994 females. Participants were stratified into two groups based on FEV1 Z-scores: those with normal pulmonary function (n = 3790) and those with pulmonary ventilatory dysfunction (n = 332). As shown in Table 1, the ventilatory dysfunction group exhibited significantly higher mean age compared to the normal pulmonary function group (54.5 vs. 43, *P* < 0.001). The ventilatory dysfunction group also demonstrated a higher proportion of males (63.25 vs. 50.61%) and higher smoking rates (62.62 vs. 43.59%). Serum concentrations of PBDE47, PBDE85, PBDE99, PBDE154, and PBB153 were significantly higher in the ventilatory dysfunction group than in the normal pulmonary function group. Additionally, significant differences were observed between the two groups in terms of race, BMI, FIR, history of hypertension, diabetes, smoking, FVC, FEV1/FVC, PEF, and FEF25–75%. Conversely, FeNO, an indicator of airway inflammation, did not show significant differences between the two groups.

**Table 1:** Characteristics of the study participants.

Variable	Total (n = 4122)	Normal pulmonary function (n = 3790)	Ventilatory dysfunction (n = 332)	*P* value
Exposures [pg/g, mean (Q1, Q3)]				
PBDE17	1.27 (0.92–1.80)	1.27 (0.92–1.80)	1.27 (0.92–1.80)	0.146
PBDE28	7.51 (5.30–11.00)	7.48 (5.30–11.00)	7.81 (5.36–11.38)	0.229
PBDE47	134.90 (92.15–205.80)	134.30 (91.60–205.50)	155.20 (100.70–214.40)	0.002
PBDE66	1.56 (1.53–1.80)	1.56 (1.50–1.80)	1.56 (1.56–1.80)	0.229
PBDE85	2.70 (1.79–4.30)	2.68 (1.79–4.26)	3.02 (1.80–4.92)	0.003
PBDE99	26.67 (17.46–43.92)	26.20 (17.46–42.50)	30.04 (17.71–51.90)	0.002
PBDE100	27.50 (18.09–42.32)	27.06 (18.04–41.70)	30.26 (19.09–45.23)	0.014
PBDE153	50.97 (34.17–90.11)	50.97 (34.39–90.11)	49.97 (33.31–90.81)	0.935
PBDE154	2.52 (1.80–4.10)	2.51 (1.79–4.07)	2.92 (1.80–4.48)	0.002
PBDE183	1.80 (1.15–1.98)	1.80 (1.14–1.96)	1.80 (1.30–2.54)	0.001
PBDE209	17.60 (13.41–19.97)	17.60 (13.40–19.71)	17.66 (14.21–22.92)	0.231
PBB153	15.25 (6.46–30.20)	14.41 (6.19–29.66)	22.24 (11.09–39.40)	< .001
Age [years, mean (Q1, Q3)]	44.00 (30.00–59.00)	43.00 (30.00–57.00)	54.50 (38.00–65.00)	< .001
FIR [mean (Q1, Q3)]	2.19 (1.11–4.22)	2.22 (1.12–4.31)	1.75 (0.91–3.44)	< .001
BMI [(kg/m^2^, mean (Q1, Q3)]	27.62 (24.10–32.13)	27.50 (24.08–31.84)	29.58 (24.53–36.00)	< .001
FeNO [ppb, mean (Q1, Q3)]	13.50 (8.50–21.00)	13.50 (8.50–20.50)	13.50 (8.50–23.12)	0.698
FVC [ml, mean (Q1, Q3)]	3898.00 (3200.25–4697.75)	3976.00 (3278.00–4774.50)	3023.50 (2321.75–3787.25)	< .001
FEV1 [ml, mean (Q1, Q3)]	3089.00 (2509.25–3738.75)	3167.00 (2598.25–3803.75)	1994.00 (1483.00–2550.50)	< .001
FEV1/FVC [mean (Q1, Q3)]	0.80 (0.74–0.84)	0.80 (0.75–0.85)	0.70 (0.59–0.78)	< .001
PEF [ml/s, mean (Q1, Q3)]	8023.00 (6656.00–9700.75)	8201.00 (6845.50–9850.75)	5690.50 (4211.75–7102.75)	< .001
FEF25–75% [ml/s, mean (Q1, Q3)]	2974.50 (2067.50–3879.00)	3083.50 (2223.25–3960.50)	1153.50 (660.75–2093.50)	< .001
Gender (%)				< .001
Male	2128 (51.63)	1918 (50.61)	210 (63.25)	
Female	1994 (48.37)	1872 (49.39)	122 (36.75)	
Race (%)				< .001
Mexican American	668 (16.21)	651 (17.18)	17 (5.12)	
Other Hispanic	458 (11.11)	442 (11.66)	16 (4.82)	
Non-Hispanic White	1794 (43.52)	1688 (44.54)	106 (31.93)	
Non-Hispanic Black	838 (20.33)	684 (18.05)	154 (46.39)	
Other Race	364 (8.83)	325 (8.58)	39 (11.75)	
Alcohol (%)				0.290
No	911 (24.46)	827 (24.23)	84 (26.92)	
Yes	2814 (75.54)	2586 (75.77)	228 (73.08)	
Hypertension (%)				< .001
No	2929 (71.13)	2752 (72.69)	177 (53.31)	
Yes	1189 (28.87)	1034 (27.31)	155 (46.69)	
Diabetes (%)				< .001
No	3651 (88.62)	3395 (89.63)	256 (77.11)	
Yes	394 (9.56)	325 (8.58)	69 (20.78)	
Borderline	75 (1.82)	68 (1.80)	7 (2.11)	
Smoke status (%)				< .001
No	2144 (54.85)	2024 (56.41)	120 (37.38)	
Yes	1765 (45.15)	1564 (43.59)	201 (62.62)	

### Correlation between individual BFRs exposure and pulmonary ventilatory dysfunction

Survey-weighted generalized logistic regression models were employed to analyze the correlation between individual BFRs exposure and pulmonary ventilatory dysfunction. After adjusting for gender and age, PBDE47 (OR: 1.861, 95% CI 1.075–3.070; *P* = 0.027), PBDE85 (OR: 1.713, 95% CI 1.093–2.684; *P* = 0.020), PBDE99 (OR: 1.592, 95% CI 1.041–2.502; *P* = 0.044), PBDE154 (OR: 1.602, 95% CI 1.035–2.479; *P* = 0.035), PBDE183 (OR: 2.081, 95% CI 1.023–4.235;* P* = 0.043), and PBDE209 (OR: 2.150, 95% CI 1.226–3.771; *P* = 0.009) showed a significant positive correlation with impaired ventilation. Even after adjusting for all covariates, PBDE47 (OR: 1.837, 95% CI 0.987–3.405; *P* = 0.055), PBDE183 (OR: 2.481, 95% CI 1.113–5.534; *P* = 0.027), and PBDE209 (OR: 1.853, 95% CI 1.063–3.229; *P* = 0.030) remained positively associated with impaired ventilation (Table 2).

**Table 2:** Association between log-transformed serum BFRs and pulmonary ventilatory dysfunction.

	Model 1		Model 2		Model 3		Model 4	
	OR (95%CI)	*P* value	OR (95%CI)	*P* value	OR (95%CI)	*P* value	OR (95%CI)	*P* value
PBDE17	2.392 (0.803, 7.126)	0.115	1.826 (0.583, 5.714)	0.294	1.807 (0.503, 6.492)	0.356	1.874 (0.548, 6.402)	0.308
PBDE28	2.612 (1.669, 4.086)	0.000	1.372 (0.776, 2.424)	0.269	1.757 (0.902, 3.422)	0.096	1.679 (0.827, 3.407)	0.147
PBDE47	2.574 (1.632, 4.061)	0.000	1.816 (1.075, 3.070)	0.027	1.899 (1.057, 3.412)	0.033	1.834 (0.987, 3.405)	0.055
PBDE66	2.958 (1.387, 6.309)	0.006	2.005 (0.910, 4.421)	0.083	2.140 (0.829, 5.528)	0.113	2.077 (0.824, 5.239)	0.118
PBDE85	2.167 (1.468, 3.200)	0.000	1.713 (1.093, 2.684)	0.020	1.710 (1.048, 2.790)	0.033	1.619 (0.972, 2.695)	0.063
PBDE99	2.038 (1.371, 3.030)	0.001	1.592 (1.014, 2.502)	0.044	1.584 (0.971, 2.584)	0.065	1.550 (0.937, 2.563)	0.086
PBDE100	1.971 (1.287, 3.019)	0.002	1.508 (0.960, 2.369)	0.074	1.497 (0.931, 2.407)	0.094	1.455 (0.875, 2.420)	0.144
PBDE153	0.961 (0.589, 1.567)	0.871	0.815 (0.519, 1.277)	0.364	1.145 (0.704, 1.862)	0.577	1.073 (0.634, 1.816)	0.787
PBDE154	2.136 (1.440, 3.170)	0.000	1.602 (1.035, 2.479)	0.035	1.508 (0.934, 2.434)	0.091	1.478 (0.908, 2.408)	0.113
PBDE183	3.092 (1.687, 5.669)	0.000	2.081 (1.023, 4.235)	0.043	2.379 (1.081, 5.236)	0.032	2.481 (1.113, 5.534)	0.027
PBDE209	2.795 (1.555, 5.022)	0.001	2.150 (1.226, 3.771)	0.009	1.816 (1.040, 3.170)	0.036	1.853 (1.063, 3.229)	0.030
PBB153	2.022 (1.456, 2.808)	0.000	1.020 (0.671, 1.551)	0.923	1.291 (0.862, 1.934)	0.209	1.277 (0.840, 1.940)	0.244

Model 1: no adjustment for any covariates, Model 2: controlled for age and gender, Model 3: additionally adjusted for race, FIR, and BMI on Model 2, Model 4: further adjusted for baseline medical history and smoking/alcohol history on Model 3. 95%CI, Confidence Interval.

**Figure 2:**
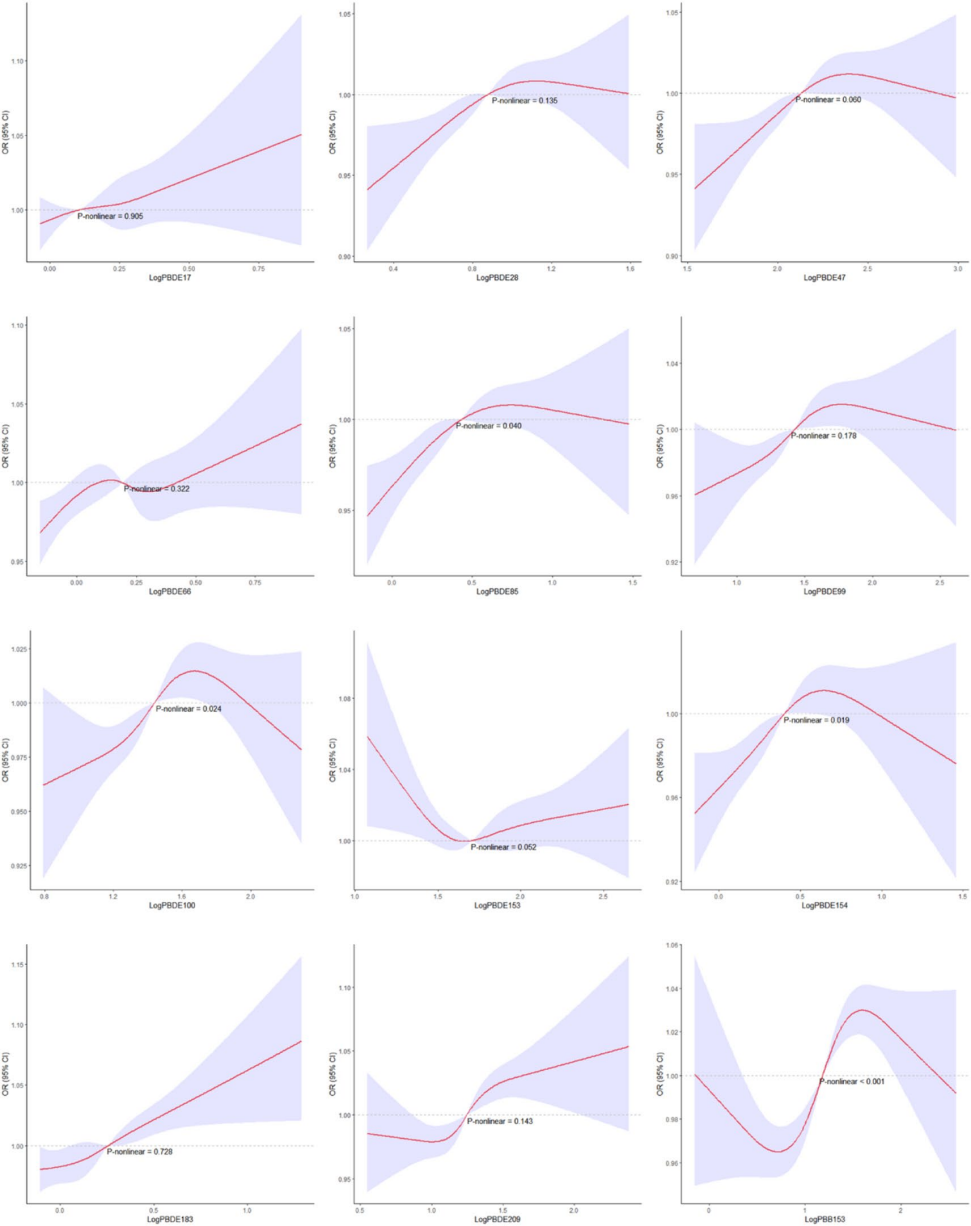
The non-linear associations between individual BFRs and pulmonary ventilatory dysfunction by restricted cubic splines.

Restricted cubic spline curves illustrated the potential nonlinear relationships between log-transformed individual BFRs concentrations and the occurrence of pulmonary ventilatory dysfunction (Fig. 2). Concentrations of PBDE85, PDBE100, and PBDE154 exhibited a reversed U-shaped relationship with pulmonary ventilatory dysfunction (*P* < 0.05), while PBB153 concentration showed an S-shaped relationship with pulmonary ventilatory dysfunction (*P* < 0.001). Furthermore, subgroup interaction analyses were performed based on gender and age. Subgroup analysis by gender revealed no interaction except for PBDE153 (Table 3). Similarly, when splitting by the median age of > 44 and ≤ 44, no interactions were observed (Table 4).

**Table 3:** Association between log-transformed serum BFRs and pulmonary ventilatory dysfunction stratified by gender.

	Subgroup	OR (95%CI)	*P* value	*P* for interaction
PBDE17	Male	1.065 (0.790, 10.666)	0.106	0.355
	Female	1.312 (0.292, 5.888)	0.718	
PBDE28	Male	2.645 (1.510, 4.649)	0.001	0.593
	Female	2.125 (1.094, 4.127)	0.027	
PBDE47	Male	2.607 (1.499, 4.533)	0.001	0.421
	Female	1.842 (0.863, 3.931)	0.112	
PBDE66	Male	3.864 (1.421, 10.508)	0.009	0.221
	Female	1.660 (0.543, 5.074)	0.366	
PBDE85	Male	2.006 (1.243, 3.239)	0.005	0.906
	Female	1.926 (1.052, 3.525)	0.034	
PBDE99	Male	2.211 (1.60, 3.596)	0.002	0.260
	Female	1.405 (0.698, 2.831)	0.334	
PBDE100	Male	1.981 (1.216, 3.228)	0.007	0.388
	Female	1.355 (0.613, 2.993)	0.445	
PBDE153	Male	1.145 (0.583, 2.248)	0.688	0.027
	Female	0.314 (0.139, 0.710)	0.006	
PBDE154	Male	2.030 (1.286, 3.205)	0.003	0.630
	Female	1.679 (0.831, 3.394)	0.145	
PBDE183	Male	2.715 (1.284, 5.744)	0.010	0.381
	Female	1.716 (0.743, 3.964)	0.201	
PBDE209	Male	2.089 (0.919, 4.752)	0.078	0.534
	Female	2.911 (1.396, 6.072)	0.005	
PBB153	Male	1.677 (1.192, 2.361)	0.004	0.182
	Female	2.246 (1.422, 3.547)	0.001

**Table 4:** Association between log-transformed serum BFRs and pulmonary ventilatory dysfunction stratified by age.

	Subgroup	OR (95%CI)	*P*	*P* for interaction
PBDE17	<= 44	2.069 (0.571, 7.490)	0.262	0.978
	> 44	2.023 (0.542, 7.554)	0.288	
PBDE28	<= 44	1.737 (0.873, 3.459)	0.113	0.864
	> 44	1.610 (0.802, 3.233)	0.176	
PBDE47	<= 44	2.922 (1.505, 5.674)	0.002	0.263
	> 44	1.886 (1.058, 3.364)	0.032	
PBDE66	<= 44	3.801 (1.566, 9.223)	0.004	0.196
	> 44	1.824 (0.720, 4.622)	0.200	
PBDE85	<= 44	2.663 (1.449, 4.894)	0.002	0.140
	> 44	1.605 (0.993, 2.595)	0.053	
PBDE99	<= 44	2.670 (1.557, 4.578)	0.001	0.077
	> 44	1.481 (0.890, 2.461)	0.127	
PBDE100	<= 44	2.820 (1.238, 6.234)	0.012	0.137
	> 44	1.441 (0.872, 2.384)	0.150	
PBDE153	<= 44	1.848 (0.646, 5.283)	0.246	0.203
	> 44	0.788 (0.420, 1.478)	0.449	
PBDE154	<= 44	2.742 (1.428, 5.266)	0.003	0.130
	> 44	1.515 (0.914, 2.511)	0.105	
PBDE183	<= 44	2.157 (0.737, 6.314)	0.157	0.605
	> 44	3.002 (1.341, 6.720)	0.009	
PBDE209	<= 44	5.211 (2.201, 1.335)	0.000	0.093
	> 44	1.848 (0.820, 4.161)	0.135	
PBB153	<= 44	1.282 (0.683, 2.405)	0.432	0.827
	> 44	1.388 (0.842, 2.289)	0.193	

### Correlation between mixed BFRs exposure and pulmonary ventilatory dysfunction

**Figure 3:**
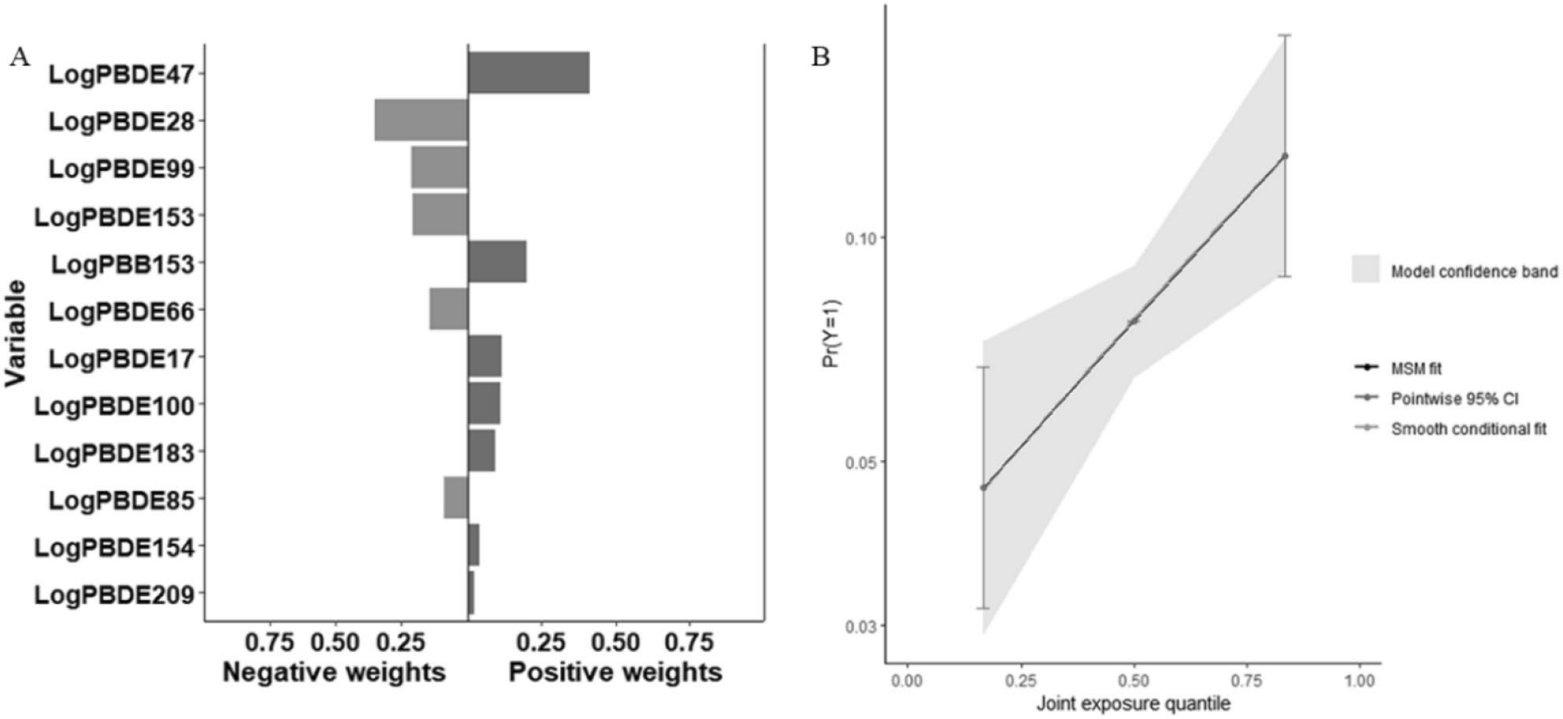
Associations of mixture BFRs exposure with pulmonary ventilatory dysfunction in all participants. (**A**) QGC regression index for ventilation dysfunction. (**B**) Joint effect (95% CI) for ventilation dysfunction in QGC analysis.

QGC analysis was utilized to examine the correlation between mixed BFRs exposure and pulmonary ventilatory dysfunction. The results indicated a positive association between mixed BFRs exposure and pulmonary ventilatory dysfunction (OR: 1.432; 95% CI 1.117–1.835; *P* = 0.005), with PBDE47 contributing the most at 41.3% (Fig. 3).

## Discussion

This study investigated for the first time the impact of BFRs on lung function in adults in the United States. The findings revealed a positive correlation between the concentrations of PBDE47, PBDE183, PBDE209, and the occurrence of impaired lung function. Additionally, a non-linear relationship, specifically an inverted U-shaped relationship, was observed between the concentrations of PBDE85, PBDE100, PBDE154, and impaired lung function. Similarly, a pattern resembling an S-shaped relationship was found between PBB153 concentration and decreased lung function. The results from the QCG analysis demonstrated that mixed BFRs exposure was also positively associated with impaired lung function. These compelling findings provide evidence for the detrimental effects of BFRs exposure on lung function and emphasize the importance of further research into the potential health consequences of these compounds.

Currently, research on the impact of BFRs on the respiratory system is quite limited. Studies at the cellular level have indicated that PBDE47, PBDE209, and PBDE99 can induce oxidative stress and inflammatory responses in bronchial epithelial cells, activating DNA damage and cell apoptosis^19,23^. In animal experiments, PBDE47 and PBDE209 have been confirmed to induce oxidative stress and cell apoptosis^40,41^, and these pathological changes are closely associated with the development of impaired lung ventilatory function. In a cross-sectional study on the COPD population, a positive correlation was found between the logarithmically transformed continuous serum levels of PBDE-28, PBDE-47, PBDE-85, PBDE-99, PBDE-100, PBDE-154, PBDE-183, and PBB-153, and the prevalence of COPD^42^. However, the results of this current study indicate a positive correlation between PBDE47, PBDE183, PBDE209, and the occurrence of impaired lung ventilatory function, while PBDE-28, PBDE-85, PBDE-99, PBDE-100, PBDE-154, and PBB-153 showed no statistically significant association with impaired lung ventilatory function. This could possibly be attributed to the heterogeneity of the study population, and further in-depth research is needed to explore specific mechanisms in the future.

The complexity of BFRs in the environment has posed challenges to previous studies, which were limited by traditional analytical strategies that focused on modeling individual chemicals separately. This approach may lead to biased estimations that do not accurately reflect the true impact of BFRs on human health and the environment^43–46^. The mixture toxicity of BFRs can significantly differ from their individual toxicities. When two or more BFRs are combined, they may exhibit antagonistic effects, reducing toxicity, or synergistic effects, enhancing toxicity. Therefore, including all the relevant chemicals in a general linear regression model or simply summing them up is not appropriate, as high correlations and extreme distributions among the chemicals can distort the results^47,48^. In this study, QGC analysis was employed to comprehensively assess the overall impact of BFRs exposure on lung function. QGC is an intuitive and computationally efficient method that estimates the associations between multiple exposure factors and desired health outcomes. Qgcomp combines the simplicity of weighted quantile and G calculations without assuming uniformity, linearity, or additivity of exposure. The QGC analysis in this study revealed a positive correlation between mixed BFRs and impaired lung function, with PBDE47 contributing the most.

Furthermore, the dose–response patterns between BFRs and impaired lung function observed in this study are consistent with previous research, which mostly show inverted U-shaped or S-shaped relationships rather than simple linear associations^43,49,50^. In a study examining the association between BFRs and metabolic disorders, it was found that BFRs, as endocrine disruptors, may exhibit different dose–response curves under different exposure distributions in epidemiological research^51^. Another study also indicated that the inverted U-shaped association between persistent organic pollutants and type 2 diabetes and metabolic syndrome may be more pronounced in younger participants, as the biological responses of physiological systems decrease with age^49,52^. This study included a population ranging from 18 to 79 years old, covering a broader range, which may result in various nonlinear relationships.

This study has several notable strengths. Firstly, there is currently limited data on population-based cohort studies specifically focusing on the effects of BFRs, particularly on respiratory outcomes. The data for this study were obtained from a large, nationally representative sample of adults in the United States, enhancing the generalizability of our findings. Secondly, this study utilized Z-scores for FEV1 instead of traditional FEV1 predicted% values, which helps to mitigate the influence of factors such as age and race on lung function classification. Additionally, we examined not only the individual effects of specific BFRs on lung function but also the overall relationship between BFRs and lung function. However, there were some limitations in our study. Firstly, being a cross-sectional study, it does not establish causality. Secondly, the sample size in this study is still relatively small, which could potentially impact the results. Thirdly, although we accounted for as many confounding variables as possible, there may still be unconsidered confounders that could influence the results. Therefore, large-scale and longitudinal studies are warranted in the future to elucidate the causal relationship between BFR exposure and respiratory impairment.

## Conclusion

This study confirms that individual and combined BFRs exposure leads to decreased ventilatory function, impacting lung function. Further validation of these results requires large-scale longitudinal studies involving broader population groups in the future.

### Supplementary Information

Supplementary Information.

## Data Availability

This study is based on data from the National Health and Nutrition Examination Survey (NHANES) (https://www.cdc.gov/nchs/nhanes). NHANES is a national survey conducted by the US Centers for Disease Control and Prevention (NCHS), which guarantees the reliability of the data. The raw data is available in the Supplemental Files.

## Abbreviations

BFRs: Brominated flame retardants
WHO: World Health Organization
QGC analysis: Quantile g-computation analysis
COPD: Chronic obstructive pulmonary disease
FeNO: Fractional exhaled nitric oxide
NHANES: National Health and Nutrition Examination Survey
FIR: Family poverty income ratio
NO: Nitric oxide
NCHS: Centers for Disease Control and Prevention
GAPS: Global Atmospheric Passive Sampling
RCS: Restricted cubic spline
PBDEs: Polybrominated diphenyl ethers
PBB-153: 2,2′,4,4′,5,5′-Hexabromobiphenyl
ATS: American Thoracic Society
NIOSH: National Institute for Occupational Safety and Health
FEV1: Forced expiratory volume in the first second
FVC: Forced vital capacity
PEF: Peak expiratory flow
FEF25–75%: Forced expiratory flow at 25–75% of FVC
FEV1/FVC: The ratio of FEV1 to FVC
BMI: Body mass index
ppb: Parts per billion
ROS: Reactive oxygen species
TLR4: Toll-like receptor 4 (TLR4)
